# Parenting the Exceptional Social-Emotional Needs of Gifted and Talented Children: What Do We Know?

**DOI:** 10.3390/children8110953

**Published:** 2021-10-22

**Authors:** Dimitrios Papadopoulos

**Affiliations:** Department of Psychology, University of Crete, 74100 Rethymno, Greece; d.papadopoulos@uoc.gr

**Keywords:** parenting gifted and talented children, giftedness, social-emotional development, exceptional needs, twice-exceptional children

## Abstract

Parenting gifted and talented (G/T) children is a journey with unique experiences that can differ from the lived experiences of parents raising non-gifted and talented children. These unique experiences typically raise concerns, influence decisions, and exacerbate stress and anxiety regarding the children’s future development and education. The purpose of this paper is to provide an overview of the current literature in order to highlight the topic of parenting G/T children with a focus on their exceptional social-emotional needs. Studies support the conclusion that significant determinants of G/T children’s personal growth are authoritative parenting, which provides autonomy and self-motivation, and parents’ behaviors and attitudes toward the exceptional needs of G/T children. Conversely, authoritarian parenting negatively impacts children’s well-being and mental health, impeding the positive development of the child’s potential. Enhancing the caregiving capacity of family members—by reducing the stress associated with their parenting and caregiving roles—can have a powerful impact on the developmental trajectory of gifted children.

## 1. Introduction

Parenting a gifted child is a journey with unique experiences that can differ from the lived experiences of parents raising non-gifted and talented (G/T) children. These unique experiences typically raise concerns, influence decisions, and exacerbate stress and anxiety regarding the future development and education of gifted children [1]. This is especially true when a child’s ability is different from that of his or her parents. Research indicates that ecosystem moderators—including parents, family, and the school environment—are critical psychosocial variables in the transformation of a child’s ability into extraordinary achievement and a successful future [2,3]. Indeed, parents can have a significant impact on their child’s development, particularly during early childhood, through everyday interactions, support, and inspiration, by providing an atmosphere that promotes the child’s later outstanding outcomes [4].

There is a lack of consensus on the conceptual framework defining giftedness [5], including the characteristics of various areas of giftedness and the developmental process in which giftedness occurs, as well as the assessment criteria for identifying giftedness (see Table 1 for a summary of the major areas of giftedness in children). Additionally, many educational systems worldwide are underdeveloped to meet the unique academic and socio-emotional needs of gifted children, which are usually not satisfied [6]. Given this reality, parents of many gifted children tend to take a highly active role in making educational decisions and solving problems related to school placement and psychosocial adjustment of their children [7]. Consequently, when combined with the unique characteristics of gifted children, such as asynchronous development, increased emotional sensitivity, and a tendency toward negative perfectionism, parenting gifted children becomes considerably more difficult. That is, there is additional pressure on parents, who feel an urgent need to respond appropriately to the inner experiences of their child.

Interest in parenting gifted children was initially expressed by Terman (1925) [8] and Hollingworth (1930) [9], who gathered some of the first data in an attempt to better understand the contribution of parents to the development of gifted children. Later studies, which were conducted predominately in the U.S., supported the notion that parents and family systems influence individuals’ talent development [10,11]. More recently, Jolly & Matthews’ (2012) [1] review supported the crucial influence of parents on the gifted child’s academic success and achievement, while other researchers [12] have illustrated the pivotal role of parents in gifted children’s social-emotional growth, particularly during critical childhood years. However, over a century of work, remarkably little progress has been made in this area, especially in low-income countries, where gifted education and childcare provision are still lacking.

The purpose of this article is to provide an overview of the current literature in the field of parenting and giftedness and discuss the critical influence of parents on gifted children’s personal growth. In addition, this review aims to be a source of knowledge for researchers and practitioners who are not experts in the field, but who are interested in learning more regarding parenting G/T children, including children with twice-exceptional characteristics. Throughout this article, the term “gifted and talented children” (G/T children) refers to students, children, or youth identified by a professional (e.g., psychologist) as demonstrating outstanding ability and competence in areas such as intellectual, creative, artistic, or leadership capacity, or in specific academic fields [13]; moreover, these children require special services or extra curriculum activities not ordinarily provided by schools in order to meet those unique needs and to fully develop those capabilities. In this framework, “twice-exceptional children”—also known as 2e—refers to gifted students who show both evidence of exceptional gifts in at least one particular domain and inconsistent academic performance because of developmental, learning, or socio-emotional challenges, which typically reduce their academic achievements in school [12].

## 2. Exceptional Needs of G/T Children: Social and Emotional Considerations

Gifted children’s social and emotional growth is as important as their intellectual development as such growth provides individuals with the skills needed to experience, cope with, and efficiently manage the unique challenges they face when interacting with others [2]. Empirical research has provided considerable controversy and mixed outcomes, thus reflecting a dichotomy between psychosocial resilience and emotional vulnerability of G/T children and their same-age peers, as well as between gifted children and their peers of the same level of intellectual development. For example, studies conducted by Terman (1925) [8] and Hollingworth (1930) [9] successfully challenged the prevailing false beliefs and traditional assumptions regarding the psychosocial progress of gifted persons and their unique characteristics. These misconceptions include the notions that giftedness is linked to hereditary deficiencies, that it is a cause of maladjusted behavior, and that it is even a neurotic disorder [2]. However, Terman subsequently found that these individuals were not only intellectually superior, but also psychosocially superior, and exhibited greater emotional strength and higher productivity across their life span as compared with non-gifted persons. More recently, Pontes de França-Freitas, Del Prette and Del Prette (2014) [14] conducted an empirical study in Brazil, in which they compared the social capabilities of 269 gifted children with 125 typically developing peers, ranging between eight and 12 years of age, using a comprehensive social skills rating system as well as a background questionnaire. The results showed that, as compared to their peers, gifted children manifest elevated social competencies, including self-control, problem avoidance, and the expression of positive feelings. Additionally, Papadopoulos (2021) [15] conducted a cross-sectional study involving 108 gifted children from Greece. The results revealed that gifted participants reported high scholastic competence and good relationships with other people, including peers and mothers, when compared with non-gifted samples of the same chronological age. 

However, other studies have indicated that, although gifted children might have an IQ that is higher than that of their non-gifted peers, G/T children’s social and emotional competencies could correspond to their age level, or could even be inferior in some cases [16]. This means that gifted children incur the risk of developing emotional difficulties and problems possibly caused by the dynamic interaction between their endogenous traits of giftedness (such as asynchronous development, overexcitability, emotional intensity, and perfectionism) and the environment in which they grow. Morawska and Sanders (2008) [17] conducted an empirical study involving 211 parents of gifted children from New Zealand. These parents mentioned that their gifted children manifested both greater emotional responses and peer difficulties when compared to their typically developing peers. G/T students struggle with unique social and emotional issues linked to their nature; such situations might thwart their optimal development, necessitating additional support in order for them to cope with these stressors [18]. 

Similar findings were found in a study of 54 highly gifted elementary students conducted in the US by Gallagher (2015) [19]. This study found that giftedness does not imply enhanced psychological well-being; nor does it lead to better social outcomes, as certain behaviors linked to giftedness can be a source of punishment by others. Furthermore, gifted children who feel that they are different from other children usually display lower self-esteem, as they lack the skills required to diminish the gap that exists between their intellectual and interest levels, and those of their non-gifted peers. In addition, studies have shown that gifted individuals experience significantly more mental health issues, including anxiety [20] and mood disorders [21], when compared to their peers. However, the current literature suggests that, although gifted children can face complex socio-emotional concerns that threaten their subjective well-being, they are likely no better psychosocially adjusted than their average-ability classmates [22]. 

The exceptional needs of G/T learners are all influenced considerably by an appropriate educational environment; an appropriate educational environment is important, as these students require challenging and complex learning experiences and opportunities to reconceptualize existing knowledge and generate new knowledge through the learning process. Gifted children, particularly those with intellectual giftedness, may experience a mismatch between their ability and the challenge presented by the classroom because most curricula focus on age norms. Thus, gifted students adhering to curricula in age-appropriate classrooms may reduce their motivation for learning, resulting in poorer academic outcomes [23]. In addition, a lack of positive school experiences may foster stress and feelings of boredom, leading to a reduction in learning accomplishments. For any exceptional talent to flourish, unique needs must be met, and the rewards presented by the individual’s environment must be concrete. Equipping gifted children with learning environments in tune with their advanced abilities and interests may have several advantages [24]. In contrast, a gifted child’s vulnerability increases when their learning and psychosocial environments are inadequate. 

A total of 432 gifted elementary students from the US were involved in a retrospective study conducted by Peterson and Ray (2006) [25]. Participants reflected upon their school experiences from kindergarten to eighth grade. The children recalled that teasing over their giftedness started in kindergarten and peaked in sixth grade, a time when the sense of belonging became important. It was particularly interesting to note that parents and teachers were often unaware of this condition, as gifted children tended to hide their social weaknesses. According to the findings of this study, an estimated 16% of eighth-grade gifted students were considered bullied—a number that had grown since kindergarten. This matter is of particular concern, as being a victim usually decreases an individual’s social and academic successes. However, conforming to a group and feeling different became more critical with age, and especially with adolescence [26].

## 3. Materials and Methods

A literature search was performed to evaluate publications related to parenting G/T children. Keywords used to conduct the literature search were “parenting gifted/talented children,” “giftedness and parenting,” and “social and emotional needs of gifted/talented children.” Abstracts were read to identify inclusion criteria, and the selected publications were read entirely. The search engines and online libraries used were PsychInfo, ERIC, National Center for Biotechnology Information (NCBI), and PubMed (U.S. National Library of Medicine National Institute of Health). Acronyms such as “parenting G/T children” and “needs of G/T children” were entered as appropriate. The inclusion criteria were qualitative and quantitative studies in the English language, published after 2000, which specifically focused on parenting gifted/talented children in relation to their exceptional (social-emotional) needs. The exclusion criteria were publications written before 2000, studies that involve small size samples, for example at least 15 participants, literature reviews, and published dissertations (PhD/Masters). These procedures yielded a total of 16 publications that met the inclusion criteria and are presented in Table 2. Utilizing a content analysis methodology [27], the selected studies, which were read in full, were organized by two main thematic concepts: (a) parenting G/T children and (b) parenting twice-exceptional children. This approach has been used in previous literature reviews [1,2]. 

## 4. Results and Discussion

### 4.1. Parenting Gifted and Talented Children: The Pivotal Role of Parents and Family Environment

Parenting both G/T learners and typically developing children can be challenging, since each child has their own personality, temperament, interests, abilities, strengths, limitations, learning styles, and life experiences. Indeed, parents of gifted children may struggle to know what to do in their role as parents; this is part of their challenge. For example, parents of multiple children are well aware that parenting styles, decisions, and behaviors that are appropriate and effective for one child may be utterly inappropriate and ineffective for another; as a result, parents must evaluate a variety of parenting alternatives and approaches for each child. Consequently, parenting is intrinsically difficult because each child is unique and requires different approaches. Accordingly, parents must apply some important strategies to help their child’s needs, such as listening, accepting, and affirming the child’s interest or passions, supplementing their learning at school with extra activities, and providing each child with age-appropriate choices and opportunities. Table 3 summarizes some strategies that parents of gifted children can apply in both home and school situations.

In terms of environmental factors, both one’s family and parents represent important ecosystem moderators of talent development and children’s personal growth [34,43], and parenting has been shown to influence children’s psychosocial adjustment since the child’s social skills are heavily shaped by an appropriate parent–child relationship [42]. Campbell and Verna (2007) [32] found that parents can develop a set of effective parenting components that can influence children’s achievement and success. These components include making appropriate decisions regarding the child’s schooling, promoting flexibility, fostering and motivating their child’s goals and attitudes toward achievement, and encouraging and engaging cooperation between home and school. Garn, Matthews, and Jolly (2010) [34] conducted a qualitative study in the United States involving 30 parents of G/T children. Results indicated that parenting with a focus on autonomy and control established an academic motivation environment at home. This, in turn, further enhanced children’s optimal learning and outstanding performance in an academic field. 

Prior work has emphasized that giftedness itself may be a stressful condition, potentially leading to changing family dynamics and causing anxiety among parents—particularly in relation to challenges based on their perceptions of having a child who is “different” and not “normal” [44]. In the case of giftedness, even if a child’s deviation is considered superior, and not a disability, parents often report negative emotions because they feel unable to offer appropriate guidance or successfully manage challenging behaviors. Additionally, parents of gifted children have few opportunities to express their feelings and concerns with others, as parents of non-G/T children may not understand, respond to, or even believe [their] parenting experiences [45]. Indeed, some parents may be scared by their child’s superiority and overwhelmed by their child’s potential. Other parents might be underprepared for their role as a parent to support the gifted child’s exceptional needs [46] and might struggle to find resources and assistance with aspects of parenting. As a result, adequate information resources, as well as social support from interpersonal relationships and support groups, are especially vital to promote both parental self-efficacy and the use of effective parenting practices which, in turn, can have a positive impact on G/T children’s successful personal growth and well-being. 

For example, in such parental support groups, parents of G/T children can find a safe place to discuss and to share stories about their children and hear experiences from other parents; moreover, they can find comfort and can learn alternative strategies for managing their child’s behavioral problems when they talk with other parents who are experiencing the same daily joys and frustrations of raising a gifted child [2,12]. In particular, the SENG (Supporting Emotional Needs of the Gifted) model [7] has been used extensively to connect parents of gifted children and support them in dealing with G/T children’s social-emotional characteristics, such as intense behavioral reactions, uneven development, and perfectionism. This model focuses on discussions among 10 to 20 interested parents of G/T children on topics such as motivation, discipline, stress management, peer relationships, and depression, and provides a non-judgmental and nurturing climate among the members of the group. However, no empirical research to date has assessed the effectiveness of this parenting program. In general, parental support groups can provide parents with sufficient information regarding meeting their child’s needs, as most parents lack a framework for understanding the developmental issues affecting a gifted child [17]. Furthermore, such groups can help educate parents about their unique role as parents of gifted children, and increase parents’ understanding of what is known and what it means to be gifted, and how it is best served. Parents’ group meetings can detail how to effectively assist G/T children from early childhood through adolescence, not just for the children’s benefit, but also for the greater good of the world in which they will develop and become productive adults [7]. The size of parents’ support networks can be increased by using both formal and informal sources of social support, such as professional-based assistance, groups of parents with similar concerns, and online communities (e.g., Facebook). Such support can help improve parenting skills and reduce parents’ anxiety. In turn, gifted children obtain benefits when their parents understand and are actively involved as advocates in all aspects of their children’s lives [40]. 

Morawska and Sanders (2009) [33] examined the efficacy of a behavioral-based intervention among 75 parents of gifted children. The results revealed that parents reported significant improvement in their own parenting styles; in addition, these changes had a positive impact on their children’s behavioral adjustment. The findings of the above study confirm that parents of gifted and talented children have unique needs in terms of parenting, and as such, parenting groups may have an impact on both gifted children’s and their parents’ social and emotional needs. However, parents raising G/T children may struggle to obtain support because they erroneously believe that support groups are unfit for their needs; however, in such groups, they can safely share experiences and concerns with other parents who face common issues, thereby reducing their anxiety. 

In an attempt to normalize their G/T child’s unique characteristics and needs and their own parenting experience—particularly in societies where giftedness is connected with disadvantages and engenders negative social reactions —parents may consciously or subconsciously deny the child’s high abilities and talents [47] (p. 399). Such parenting experiences have been associated with profound guilt [46], which explains why, despite the joy of having a gifted child, these parents may feel nervous, overwhelmed, and experience a burden of parenting. Consequently, such emotions may, directly and indirectly, impact gifted individuals’ self-worth and social development, thereby affecting the entire family life and dynamics. 

For parents of G/T children, choosing an appropriate school can also be a burden, causing stress and frustration. This is particularly true when parents’ dissatisfaction with the curriculum can lead to a decision to change their child’s school or choose to homeschool their gifted child based on the belief that the curriculum is insufficiently developing appropriate academic opportunities [7]. This, in turn, can be a source of external risk that influences children’s academic outcomes and passion for learning, and may affect their socialization. Additionally, parents usually experience frustration directed toward teachers and administrators within the school system when teachers misunderstand their gifted child’s behavior and when programming for gifted children does not fit the academic needs of their children, and when academic options available to their children are limited [30]. Research has also suggested that when parents have unreasonable expectations about their children’s academic skills and success, this can be a risk factor for gifted children to develop emotional and behavioral difficulties, such as anxiety and poor peer relations, as well as difficulties with managing academic failure [33].

A body of research suggests that families of gifted children can be characterized as resilient [29,48]. Members of these families have clear roles and warm relationships and exhibit satisfactory levels of cohesion and bonding, which contribute to optimal socio-emotional growth and well-being [38]. Cornell and Grossberg (1987) [49] found that parents of gifted children reported themselves as being more cohesive and expressive, and less controlling than parents of nongifted children. Cohesion and expressiveness were correlated with fewer conduct problems, less anxiety, higher self-esteem, and greater academic performance in gifted children. For gifted children, parents’ warm expressiveness, authoritative parenting, and harmonious family relationships are positively linked with higher levels of self-esteem and well-being, as well as lower levels of mental health problems [29]. Furthermore, the parents of such families emphasize an unconditional positive regard for their children and encourage their independence and self-motivation.

Yazdani and Daryei (2016) [39] examined the impact of parenting styles on the psychosocial adjustment of 118 gifted adolescents. Consistent with previous studies, they found that an authoritative parenting style was associated with teenagers’ positive socio-emotional adjustment, while an authoritarian parenting style may negatively impact gifted teens’ mental health and wellbeing. Additionally, Olszewski-Kubilius (2002) [50] asserted that G/T children are helped to achieve optimal adjustment by parents who provide emotional support and bonding and give children “psychological space,” which allows them to encounter and cope with challenges. Likewise, research has highlighted that parental (and particularly maternal) acceptance is positively associated with children’s creativity and can influence both the child’s academic and social-emotional development during early childhood [1,15]. Indeed, building a warm parent–child relationship is critical for establishing secure attachments, which, in turn, allows children to build trust and favorable self-esteem. In contrast with previous studies, Pilarinos and Solomon (2017) [42] found an interesting, but paradoxical finding: mothers’ authoritative parenting style was associated with negative outcomes in gifted children, including more conduct and behavioral problems, while authoritarian parenting was linked to gifted children perceiving themselves as more intelligent and successful at school. 

The parental behaviors identified by previous research can be categorized into parenting styles as supported in Baumrind’s (1967; 1971) [51,52] standard typology, which includes three main styles of parenting behavior: authoritarian, permissive, and authoritative. Authoritarian parents tend to show high demandingness and low responsiveness regarding their children’s behavior and needs. For example, such parents set strict rules and standards that the child must obey, and typically have high expectations of the child. Meanwhile, these parents show little physical or verbal responsiveness and expressiveness toward their children. In contrast, permissive parents are characterized by exerting less control over their child’s choices, even if this is not developmentally appropriate. Further, permissive parents are communicative, warm, and behave more as a coach than an ultimate authority figure. Between these two extreme parenting styles, an authoritative parent directs the child’s behavior via a set of family rules and standards but is also flexible. Parental control is applied using strategies such as discussion, which encourages independent thinking by the child through communication and understanding. Finally, based on Maccoby and Martin‘s (1983) [53] work, Baumrind (1991) [54] introduced a fourth category: neglectful/uninvolved parents, who are characterized by low levels of both demandingness and responsiveness. 

More recently, Kuppens and Ceuleman (2019) [55] asserted that specific combinations of parenting practices within a parent, rather than distinct parenting practices or dimensions, have a greater impact on child development and behavior. Children raised by both parents in intact homes are influenced by both parents’ combined practices. Given that studies have found that mother’s and father’s parenting styles might differ, analyzing the combination of both maternal and parental practices provides a more accurate perspective on two-parent households and families [37,42]. However, the impact of joint parenting styles on a child’s social-emotional development has received little to no attention in research on giftedness, with most studies focused on Baumrind’s parenting typology. 

With regard to gender and parenting, research has provided inconsistent findings. For example, in a sample of gifted children and their parents from Canada, Pilarinos and Solomon (2017) [42] found that mothers were significantly more authoritative than fathers. This finding may be explained by gender roles in parenting and family responsibilities, as mothers tend to adopt a more flexible family caregiving role compared to fathers, who are less involved in parenting their children and primarily tend to adopt disciplinary and authoritarian roles [56]. In contrast, a study conducted by Basirion, Majid, and Jelas (2014) [37] in Malaysia revealed gifted children perceived their mothers to employ strict discipline and demands and to criticize them for any possible mistakes, more so than did fathers. The authors asserted that the different impact of fathers and mothers’ parenting styles may partly be explained by the effect of maternal depression and attachment problems on gifted children’s socio-emotional adjustment. Yang (2007) [31] focused on Chinese American parents of gifted children, finding that parents influenced their child’s achievement by exhibiting high levels of guidance and support, while they tended to use a more training type of parenting style. 

In contrast, gifted and talented children who grow up with authoritarian parents seem to experience psychological distress, and their families tend to show lower levels of connectedness and emotional closeness [1]. In addition, research has connected authoritarian styles with a greater risk of developing mental health problems, including insecure attachment style in adults [57] and lower levels of social-emotional well-being [39]. 

Furthermore, studies have shown a link between authoritarian parenting and some types of perfectionism [58]. According to Flett and colleagues (2002) [59], gifted children tend to develop maladaptive perfectionism, particularly when parental love and approval are seen as conditional on performance, and when parents respond to the child’s needs depending on their academic success. Similarly, Basirion, Majid, and Jelas (2014) [37] found relationships between unhealthy perfectionism, authoritarian parenting, and high parental expectations, as well as positive perfectionism and authoritative parenting. 

### 4.2. Parenting Twice-Exceptional Children 

Twice-exceptional (2e) learners have both exceptional ability and disability—developmental, learning, emotional, physical, or sensory—creating a unique set of circumstances. Their remarkable talent may overshadow their disability or vice versa [60]. Studies of twice-exceptional children have identified a complicated set of learning, social, behavioral, and emotional qualities that represent both academic strengths and weaknesses [61]. Identification of twice-exceptional students remains a problem because they possess the characteristics of gifted students and the characteristics of students with disabilities who struggle with many aspects of learning. Typically, 2e students have superior verbal abilities (e.g., vocabulary and advanced ideas), a wide range of interests and opinions, and are highly creative [12]. At the same time, they may exhibit hyperactivity, emotional intensity, conduct problems, and a lack of organizational skills in learning. As a result, each aspect may mask other aspects, preventing such children from being identified and considered for services for gifted persons. However, for some gifted individuals, their disability is recognized, but its severity masks the expression of gifts and talents and providing remedial interventions prevails over addressing academic achievement. 

The parents of twice-exceptional children experience several primary areas of concern. First are concerns associated with the abilities of school personnel (such as teachers and school psychologists) to provide appropriate academic experiences for 2e learners and to support their social-emotional development, including those who exhibit asynchronous development and experience problems with social adjustment [62]. Such concerns generally arise because most school personnel lack in both providing strengths-based opportunities and those tailored to remediate learning deficits, both of which have been found to be crucial for twice-exceptional children [63]. Consequently, tensions and conflicts frequently develop in the relations between parents and school providers; moreover, these relations are characterized by a lack of trust. Although there are few private schools for gifted and twice-exceptional children worldwide, parents of children attending such schools reported feeling more satisfied with their child’s education and becoming more actively involved in their child’s education than they did in regular public-school settings [28]. 

Second, the literature indicates that other typical concerns are parents’ continuous efforts to serve as their child’s case manager, mentor, and advocate [64]. These efforts primarily consist of seeking professional evaluations and providing or securing educational support, in spite of the inconvenience and economic cost. Indeed, parents’ education and their ability to provide energy and financial support to search for professional assistance with their child’s needs are significant factors in the outcomes of twice-exceptional learners. To address the specific demands of parenting 2e students, parents must maintain high expectations for their children, despite the child’s disabilities, and be involved in their education—very often beyond what is provided within the school environment [36]. The quality of participation is likely to affect both the academic progress and social and emotional functioning of the students [65]. Furthermore, supporting the unique needs of twice-exceptional learners necessitates collaboration among all stakeholders, including students, parents, school professionals, and policymakers. Without this type of teamwork, a student’s tailored educational program is unlikely to include interventions that will maximize student progress while also addressing both learning and social-emotional needs [64]. 

Parents are usually the first to notice their children’s gift(s), as well as their disabilities and, are usually in the best position to gather information about their children’s learning capabilities and limitations so that they may work with schools to acquire the best educational services at the earliest opportunity. This usually occurs as a result of their personal observations of their children, as well as via information obtained from those involved in their children’s lives, such as close family members, teachers, and school psychologists. To plan the most appropriate individualized curriculum, parents should be vocal in their advocacy efforts to ensure that educators and other school professionals, such as psychologists and counselors, have conducted a thorough academic and psychological evaluation and that they understand the 2e learners’ special needs. Additionally, parents of 2e learners can teach their children strategies by which to realize their potential and develop healthy perceptions of their strengths and disabilities [66]. Parents should be the first persons to inform teachers about their children’s weaknesses and necessary accommodations; however, it will be the children’s responsibility to advocate for themselves. 

Reis, McGuire, and Neu (2000) [67] and Reis, Neu, and McGuire (1997) [68] also focused on parents’ important role in their 2e learners’ social and emotional development and needs. They found that positive parental involvement focuses on increasing children’s self-confidence and developing their strengths, while also addressing their weaknesses is critical to the social-emotional wellness of 2e learners as they face difficulties in this area. Parents who took part in these studies encouraged their children to have positive outside-of-school experiences based on their interests, which may have helped these children to positively adapt to their negative school experiences. Support and acceptance received from their parents helped these children to realize, appreciate, and accept their capabilities outside of school, as well as to develop compensating tactics and personal strategies to excel academically.

## 5. Summary and Conclusions

This article presents an overview of the current literature on parenting G/T children and discusses the pivotal role of parents in expanding gifted children’s personal growth. However, research into differences between parenting G/T children and average-ability children is limited. For example, Robinson, Lanzi, Weinberg, Ramey, and Ramey (2002) [69] found that parents of gifted children were more responsive (e.g., caring, and interactive with their children) and nonrestrictive (e.g., encouraging their children to express their opinions) compared to parents of nongifted children. Parents of gifted children also contributed more at their child’s school and were seen as more supportive of their child’s academic endeavors by teachers. More recently, Yazdani and Daryei (2016) [39] evaluated the effects of parenting styles on 118 gifted and 115 typically developing adolescents’ adjustment. They found that parents of gifted adolescents are more authoritative and less authoritarian than are parents of non-gifted adolescents, and that gifted adolescents have a more positive attitude toward their parents than do average-ability adolescents. These findings may be related to parents’ concerns about their gifted children’s growth, which may affect the time and energy parents dedicate to providing extra-curricular home-related learning opportunities and support for their children. Accordingly, G/T children may have more chances to interact with their parents during developmentally appropriate activities and resources. Such practices can promote parent-child bonding, allowing parents to use more authoritative practices with G/T children while reducing parental stress related to the responsibilities and unique challenges of raising a precocious child.

Compared to parents of average-ability children, parents of G/T children typically recognize the complexity of their parenting role and encounter increased parenting challenges that enable them to develop strategies that guide and support their child’s exceptional needs [12]. This sense of the increased burden of responsibility in terms of parenting the child’s special needs can and often does exacerbate stress in parents of G/T children compared with other parents [40]. Literature on giftedness and parenting styles supports that parents of G/T children typically use an authoritative parenting style, which is linked to children’s optimal development [29,39,42], whereas other studies suggest that permissive and authoritarian parenting styles are not recommended for G/T children [7]. Children who are supported by their parents in the long-term process of talent development and enjoy growing up in families with authoritative parents who promote emotional stability and experiences of love are likely to become successful adults [2]. Based on Baumrind’s typology it seems that authoritative parents who demonstrate warmth, acceptance, and sensitivity, combined with high expectations, may be successful in meeting G/T children’s needs because children in such an environment feel free to question and explore, thus expanding their horizons and challenging their own and others’ thinking [35,52]. In this framework, Rudasill et al. [35] suggest that G/T children who are more cognitively able elicit authoritative parenting because their behavioral characteristics—such as autonomy, self-discovery, and curiosity as well as a strong desire to explore the environment and understand complex issues—indicate that they can cope with more independence and responsibility. Consistent with this finding, Snowden and Christian [70] reported that authoritative parents of young, gifted children encouraged their children’s creativity, showed low levels of frustration, used flexible control, demonstrated confidence in their parenting role, and helped the learning process both in school and at home. 

Each gifted child has a unique combination of personality traits and areas of giftedness, and each of these traits can manifest in both positive and negative ways. The challenge of parenting a gifted child is to help them express positive manifestations and manage the negative manifestations of gifted traits [60]. Additionally, supportive parents should not only influence the child’s view of the world but also influence the skills the child acquires to navigate the world effectively. 

Parenting a child who experiences the world in a much more intense way or who has twice the exceptionality that affects his/her actions at school and home can be both a blessing and a challenge. This overview has confirmed that gifted learners who have positive parental relationships typically exhibit higher academic achievement and more positive self-perceptions, self-motivation, and overall healthy psychosocial adjustment [42,71]. Further, the unique social-emotional needs of both gifted children and 2e learners can affect parental behavior toward the gifted child and vice versa. As parents often feel unprepared to support the child’s exceptional needs, this, in turn, can create feelings of frustration and stress for both parents and gifted children. Finally, studies suggest that parents of G/T children tend to be more authoritative and less authoritarian than parents of non-gifted children; this parenting style has a positive impact on G/T children’s achievement, social-emotional development, and well-being [39,69].

Most research studies included in this review used parental self-report to assess parenting styles; this method is vulnerable to response bias, which could have an impact on the validity and generalizability of the research findings. In contrast, the inclusion of data from independent sources, such as adolescents’ self-reported perceived parenting styles supports the validity of the observations. Because most research in this review considered the connections between parenting styles and gifted children’s social-emotional outcomes using cross-sectional designs, it is difficult to determine cause-and-effect relationships. Furthermore, generalization of research findings to all gifted people is limited due to the characteristics of the samples: middle-class families, small age ranges, over-representation of mothers, under-representation of gifted children from minority groups, participants who lived in urban areas, and a lack of consensus on the criteria by which the gifted children were identified. Therefore, more research is needed to discover the values and cultural characteristics of G/T children and their families.

Parents of both G/T and twice-exceptional learners can use a talent development lens to encourage their children’s growth in terms of academic and social-emotional needs. More specifically, parents are explicitly urged to engage their children in in-school and out-of-school activities using a strength-based, talent-focused approach, providing opportunities to develop students’ advanced skills and talents in their own right [63,72]. A talent-focused approach entails identifying and recognizing learners’ strengths and abilities, as well as developing and maturing their passion for learning, and providing enrichment opportunities that develop and broaden possible areas of talent or interest. Therefore, talent development must be recognized as a necessary and non-negotiable part of any parenting strategy for both gifted/talented and twice-exceptional children.

Given the heterogeneity of gifted populations and the diversity of parenting practices across the globe, childcare providers, teachers, and psychologists working with gifted children must be aware of the concerns of parents and families when raising a precocious child. Developing family based interventions that enhance the caregiving capacity of family members—by reducing the stress associated with their parenting and caregiving roles—can have a significant impact on the course of giftedness [73] (pp. 216–217). By providing emotional support and love, as well as careful attention, parents may provide opportunities to maximize their child’s potential and guide gifted and talented children and youth to become successful, happy, and productive adults [12]. 

## Figures and Tables

**Table 1:** Areas of giftedness in children.

Areas of Giftedness in Children		Assessment Criteria
1	General intellectual ability	A full-scale IQ score of the 95th percentile or above on at least one battery of an intelligence or cognitive test (e.g., Wechsler Intelligent Scales, Stanford–Binet Test)
2	Specific academic aptitude (reading, writing, math, science, social studies, world language)	A score of the 95th percentile or above on a norm-referenced achievement test, or on a normed observation scale for a specific academic content area, or expert juried performance (at least advanced or distinguished)
3	Specific talent aptitude (arts, music, dance, psychomotor, creativity, leadership)	A score of the 95th percentile or above on a norm-referenced creativity test or an advanced score on an approved criterion-referenced specific talent test, and/or 95th percentile or above on a normed observation scale in an area of talent

**Table 2:** Summary of literature on parenting G/T children (2000–2020).

	Reference	Country	Participants	Major Findings
1	Hishinuma (2000) [28]	U.S.	98 parents	Parents reported that a specialized school for twice-exceptional learners was more successful at serving 2e students than previous school settings.
2	Dwairy (2004) [29]	Israel	118 gifted adolescents 115 non-gifted adolescents	Gifted adolescents perceived their parents to be more authoritative. Authoritative parental style related positively to the mental health of both gifted and non-gifted adolescents. Authoritarian parenting style negatively affected the mental health of the gifted cohort.
3	Huff, Houskamp, Watkins, Stanton, & Tavegia (2005) [30]	U.S.	15 parents	Parents reported several concerns about a lack of appropriate educational opportunities for their gifted children; teachers had limited knowledge of needs specific to African American gifted students.
4	Yang (2007) [31]	U.S.	135 parents	Chinese American parents tended to focus effort toward their gifted child’s schooling and used a training-type parenting style.
5	Campbell & Verna (2007) [32]	Europe, Asia, U.S.	2866 parents 10,062 students	A set of effective parenting components provided a positive academic home climate, which promoted student achievement.
6	Morawska & Sanders (2009) [33]	Australia	75 parents	A behaviorally based intervention was implemented with 75 parents of gifted children. Parents (a) reported significant improvement in their parenting styles and (b) these changes had a positive impact on their child’s behavioral adjustment.
7	Morawska & Sanders (2008) [17]	Australia	211 parents	Parents reported that their children showed signs of emotional symptoms and peer problems. Parents used a more authoritarian parenting style characterized by lecturing.
8	Garn, Matthews, & Jolly (2010) [34]	U.S.	30 parents	Parenting with a focus on autonomy established an environment of academic motivation.
9	Rudasill, Adelson, Callahan, Houlihan, & Keizer (2013) [35]	U.S.	332 children aged 8–17 years	Highly able children were more likely to perceive their parents as adopting an authoritative parenting style. Flexible-democratic parenting style influenced the cognitive development of children. Girls found their parents to be more authoritarian than boys, while African American children found their mothers to be more authoritarian.
10	Speirs Neumeister, Yssel1, & Burney (2013) [36]	U.S.	20 primary caregivers and their children	Primary caregivers reported that they (a) had a critical role in the academic success of their twice-exceptional children, and (b) had high expectations of their children, despite their disabilities.
11	Basirion, Majid, & Jelas (2014) [37]	Malaysia	448 academically gifted adolescents	Positive perfectionism was related to parental paternal authoritative style, while negative perfectionism was significantly predicted by parental authoritarian style.
12	Olszewski-Kubilius, Lee, & Thomson (2014) [38]	U.S., and South Korea	1526 parents and their children	Gifted children rated their families as cohesive and flexible with high levels of satisfaction and communication among family members. Parents of G/T children perceived their families as cohesive and flexible, and felt positive about communication among their family members.
13	Yazdani, & Daryei (2016) [39]	Iran	118 gifted children 115 non-gifted children	Parents of gifted adolescents employed an authoritative parenting style. The perceptions of gifted adolescents towards their parents were more likely positive than those of their age-peers. An authoritative parenting style correlated positively with the psychological adjustment of both gifted and typically developing adolescents.
14	Renati & Bonfiglio (2017) [40]	Italy	49 parents	Close relatives of families of gifted children used inappropriate communication methods, which caused frustration both in children and families. Parents reported a lack of regular family routines, which is a major concern that resulted in experiencing stress.
15	Koshy, Smith & Brown (2017) [41]	U.K.	21 parents and caregivers	Parents reported concerns regarding their children’s growing up in economically deprived urban areas. Supportive parents and families providing warm communication with their G/T children was critical in increasing the motivation of both the child and the parent.
16	Pilarinos & Solomon (2017) [42]	Canada	81 parents 48 gifted children 33 teachers	Parents of gifted children described their parenting style as authoritative. Mothers’ authoritative parenting style was related to more conduct problems in gifted children. Mothers’ authoritarian parenting style was associated with gifted children perceiving themselves as more intelligent and successful at school.

**Table 3:** Parenting strategies for helping G/T children.

	Strategy	Examples
1	Communication	Listening carefully and without judgment to the child’s needs
		Encouraging the child to take sensible risks Helping the child understand what giftedness is and discuss the implications of this high-level of development Allowing for mistakes and false paths
2	Support and Encouragement	Asking questions about the way that the child is learning at school
		Providing assistance with school projects in an appropriate way
		Encouraging attention and active involvement
3	Age-appropriate responsibilities and choices	Providing after school and extra curriculum experiences based on the child’s ability level, interests, and passions
		Providing the child with a list of age-appropriate options to choose enrichment activities

## Data Availability

Results are based on public data from the included studies.
